# *N-*acetyl-*D*-glucosamine kinase binds dynein light chain roadblock 1 and promotes protein aggregate clearance

**DOI:** 10.1038/s41419-020-02862-7

**Published:** 2020-08-14

**Authors:** Md. Kamal Hossain Ripon, HyunSook Lee, Raju Dash, Ho Jin Choi, Diyah Fatimah Oktaviani, Il Soo Moon, Md. Nazmul Haque

**Affiliations:** 1 Department of Anatomy, Dongguk Medical Institute, Dongguk University College of Medicine, Gyeongju, 38066 Republic of Korea; 2 Section of Neuroscience, Dongguk Medical Institute, Dongguk University College of Medicine, Gyeongju, 38066 Republic of Korea; 3 Dongguk Medical Institute, Dongguk University College of Medicine, Gyeongju, 38066 Republic of Korea; 4 Present Address: Department of Pharmacy, Mawlana Bhashani Science and Technology University, Tangail, 1902 Bangladesh; 5 Present Address: Department of Fisheries Biology and Genetics, Patuakhali Science and Technology University, Patuakhali, 8602 Bangladesh

**Keywords:** Protein quality control, Protein aggregation, Cell death in the nervous system, Parkinson's disease

## Abstract

Emerging evidence indicates that neurodegenerative diseases (NDs) result from a failure to clear toxic protein aggregates rather than from their generation. We previously showed *N*-acetylglucosamine kinase (NAGK) promotes dynein functionality and suggested this might promote aggregate removal and effectively address proteinopathies. Here, we report NAGK interacts with dynein light chain roadblock type 1 (DYNLRB1) and efficiently suppresses mutant huntingtin (mHtt) (Q74) and α-synuclein (α-syn) A53T aggregation in mouse brain cells. A kinase-inactive NAGK_D107A_ also efficiently cleared Q74 aggregates. Yeast two-hybrid selection and in silico protein–protein docking analysis showed the small domain of NAGK (NAGK-D_S_) binds to the C-terminal of DYNLRB1. Furthermore, a small peptide derived from NAGK-D_S_ interfered with Q74 clearance. We propose binding of NAGK-D_S_ to DYNLRB1 ‘pushes up’ the tail of dynein light chain and confers momentum for inactive phi- to active open-dynein transition.

## Introduction

Excessive accumulation and aggregation of a specific protein, either a wild or mutant type, is referred to as proteinopathy^1^, which is a hallmark of many neurodegenerative diseases (NDs), including Alzheimer’s disease (AD), Parkinson’s disease (PD), and Huntington’s disease (HD)^2^. However, cells are endowed with complex surveillance machineries that identify faulty proteins in need of repair or disposal with the aid of chaperones, which act as central coordinators^3^. When a native folding state cannot be attained, chaperones typically label the misfolded protein for proteolytic removal via the ubiquitin (Ub)–proteasomal system (UPS)^4^. However, under proteotoxic stress, the capacity of the chaperone-UPS system may be swamped by the overproduction of misfolded proteins^5^. In such cases, aggregation-prone proteins not degraded may be transported along microtubules (MTs) in a retrograde manner to a microtubule organizing center (MTOC) to form an “aggresome” in the juxtanuclear region^6^. Aggresomes are then captured by lysosomes and cleared by the autophagy-lysosome pathway (ALP)^7^.

Cytoplasmic dynein is a minus end-directed microtubule motor protein and is involved in a diverse range of intracellular vesicular traffickings^8^. Dynein transports misfolded protein aggregates to MTOCs and many studies have reported direct or indirect evidence of dynein participation in NDs^9^. Although lysosomes are broadly distributed throughout cytoplasm, they concentrate in a central region surrounding the MTOC (the ‘perinuclear cloud’)^10^. Because protein aggregates might be transported by dynein motor to MTOCs to form “aggresomes”, it is reasonable to assume dynein activity increases accelerate the ALP. In this regard, *N*-acetylglucosamine kinase (NAGK) is a good candidate for promoting dynein activation. Specifically, NAGK interacts with dynein light chain roadblock type 1 (DYNLRB1)^11^ to promote dynein functions during mitosis^12^ and axonal^13^ and dendritic growth^14,15^. In the present study, we asked whether NAGK also promotes the clearance of protein aggregates. For this purpose, we tested two mutant proteins that are prone to aggregate; namely, mutant huntingtin (mHtt) with polyglutamine expansion that forms aggregates in HD and α-synuclein (α-syn) A53T that forms aggregates in PD.

## Results

### Exogenous NAGK expression suppressed mutant huntingtin (mHtt) aggregation

**Fig. 1:**
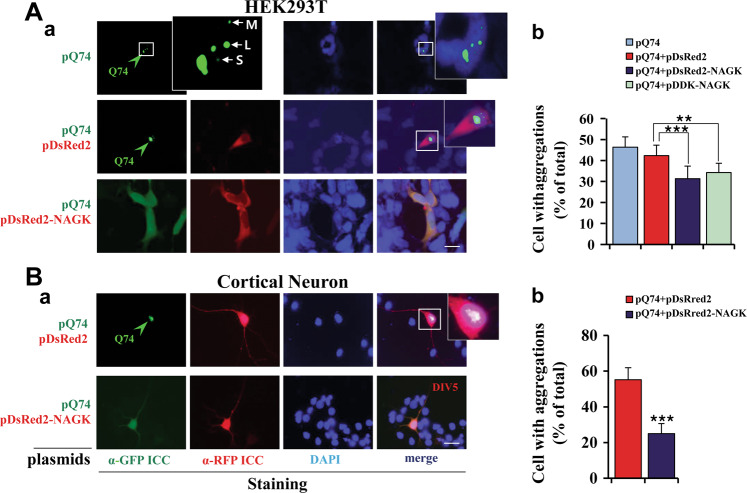
Exogenous NAGK expression suppressed the formation of Q74 aggregates in cellular HD models. **A** The HEK293T model. (a) Representative immunofluorescence images. HEK293T cells were transfected with pEGFP-Q74 (pQ74) alone (top panels) or co-transfected with pEGFP-Q74 (pQ74) and pDsRed2 (middle panels) or pDsRed2-NAGK (bottom panels). After 72 h of incubation, cells were double-labeled with indicated antibodies and then counterstained with DAPI. Q74 aggregates are marked with green arrowheads, and the boxed inset in the top panel is an enlargement showing large (L), medium (M), and small (S) aggregates (marked with arrows). (b) Cells containing Q74 aggregates were counted; results are expressed as percentages of all cells (*n* = 305). **B** The neuronal model. Rat embryonic (E19) cortical neurons were transfected on DIV 2 and incubated for 72 h. Typical images (a) and statistics (*n* = 100) (b). The annotations used are the same as those used in (a). **p < 0.01, ***p < 0.001. Scale bar = 10 µm.

We first investigated the effect of NAGK on mHtt aggregate formation in HEK293T cells (a human embryonic kidney cell-line). To facilitate aggregate formation, we transfected cells with pEGFP-Q74 (pQ74), which expresses EGFP-tagged HTT partial exon 1 (Q74) protein. At 72 h post-transfection, Q74 formed aggregates of various sizes in nuclei and cytoplasm (Fig. 1A–a, Q74). To observe the effects of NAGK on the formation of Q74 aggregates, cells were co-transfected with pQ74 and pDsRed2-NAGK (Fig. 1A–a, pQ74 + pDsRed2-NAGK). After incubation for 72 h, cultures were double-immunostained with antibodies against GFP and RFP, which revealed green fluorescent Q74 and red fluorescent DsRed2-NAGK proteins, respectively; nuclei were observed by DAPI counterstaining. Interestingly, DsRed2-NAGK expression suppressed the formation of Q74 aggregates (Fig. 1A–a, pQ74 + pDsRed2-NAGK), indicating that pDsRed2-NAGK either suppressed the formation of Q74 aggregates or efficiently promoted their clearance. The transfected cells exhibited homogeneous green fluorescence, indicating Q74 was still present in cells. Since NAGK was tagged with red fluorescent DsRed2, we also co-transfected cells with pQ74 and pDsRed2 as a control (Fig. 1A–a, pQ74 + pDsRed2) and incubated them for 72 h. DsRed2 control protein did not reduce Q74 aggregates (Fig. 1A–b). To eliminate the effect of DsRed2 protein, we co-transfected cells with pQ74 and Myc-DDK-tagged NAGK-expressing plasmid (pDDK-NAGK) (DDK is the FLAG-tag epitope with the DYKDDDDK sequence motif). As was expected, this NAGK plasmid also significantly suppressed Q74 aggregate formation. Statistical analysis (Fig. 1A–b) showed that the exogenous expressions of DsRed2-NAGK and of DDK-NAGK significantly (both *p* < 0.001) reduced the percentage of cells containing aggregates as compared with the DsRed2 control.

Next, we investigated whether the same effect occurred in cortical neurons because Huntington disease especially affects the cortex and striatum of human brain^16^. For this purpose, neurons from rat embryonic (E19) cortices were cultured and co-transfected on DIV 2 with pQ74 plus pDsRed2-NAGK or with pDsRed2 as a control. After incubation for 72 h, Q74 aggregates were rarely seen in neurons co-transfected with pQ74 and pDsRed2-NAGK (Fig. 1B–a), whereas neurons co-transfected with pQ74 and pDsRed2 contained large Q74 aggregates. Furthermore, the exogenous expression of DsRed2-NAGK significantly (*p* < 0.001) reduced the percentage of neurons containing aggregates as compared with the DsRed2 control (Fig. 1B–b).

### Exogenous NAGK expression suppressed α-syn aggregation

Next, we investigated the effects of NAGK on α-syn aggregation. For this purpose, HEK293T cells were co-transfected with pHM6-alpha-synuclein-A53T (pα-syn A53T) plus pDsRed2-NAGK or with pDsRed2 as a control. After incubation for 72 h, cultures were double-stained with anti-α-syn and -RFP antibodies and then with DAPI (Fig. S1A). Control transfection (Fig. S1A–a, pα-syn A53T + pDsRed2) revealed large aggregates in nuclear (green arrowhead) and cytoplasmic regions (white arrowheads) of transfected cells. However, when HEK293T cells were co-transfected with pα-syn A53T and pDsRed2-NAGK, most pα-syn A53T transfected cells did not exhibit such aggregations (Fig. S1A–a, pα-syn A53T + pDsRed2-NAGK). Furthermore, exogenous DsRed2-NAGK significantly (*p* < 0.01) reduced the percentage of aggregation-positive cells as compared with the DsRed2 control (Fig. S1A–b).

Next, we investigated whether similar phenomena occur in neuronal cells. For this purpose, primary rat cortical cells were co-transfected on DIV 2 with pα-syn A53T and pDsRed2-NAGK or pDsRed2. After incubation for 72 h, no aggregates were formed in neurons co-transfected with pDsRed2-NAGK, whereas many large aggregates were observed in control neurons co-transfected with pDsRed2 (Fig. S1B–a). Moreover, the percentage of aggregate-positive neurons was markedly lower (*p* < 0.01) in neurons co-transfected with pDsRed2-NAGK than with pDsRed2 (Fig. S1B–b). These results show that NAGK can suppress the formation of α-syn A53T aggregates in neuronal and non-neuronal cells and suggest aggregation suppression is a general function of NAGK.

### Short hairpin (sh) RNA or the small domain of NAGK increased Q74 aggregates, whereas kinase-inactive NAGK had a suppressive effect

**Fig. 2:**
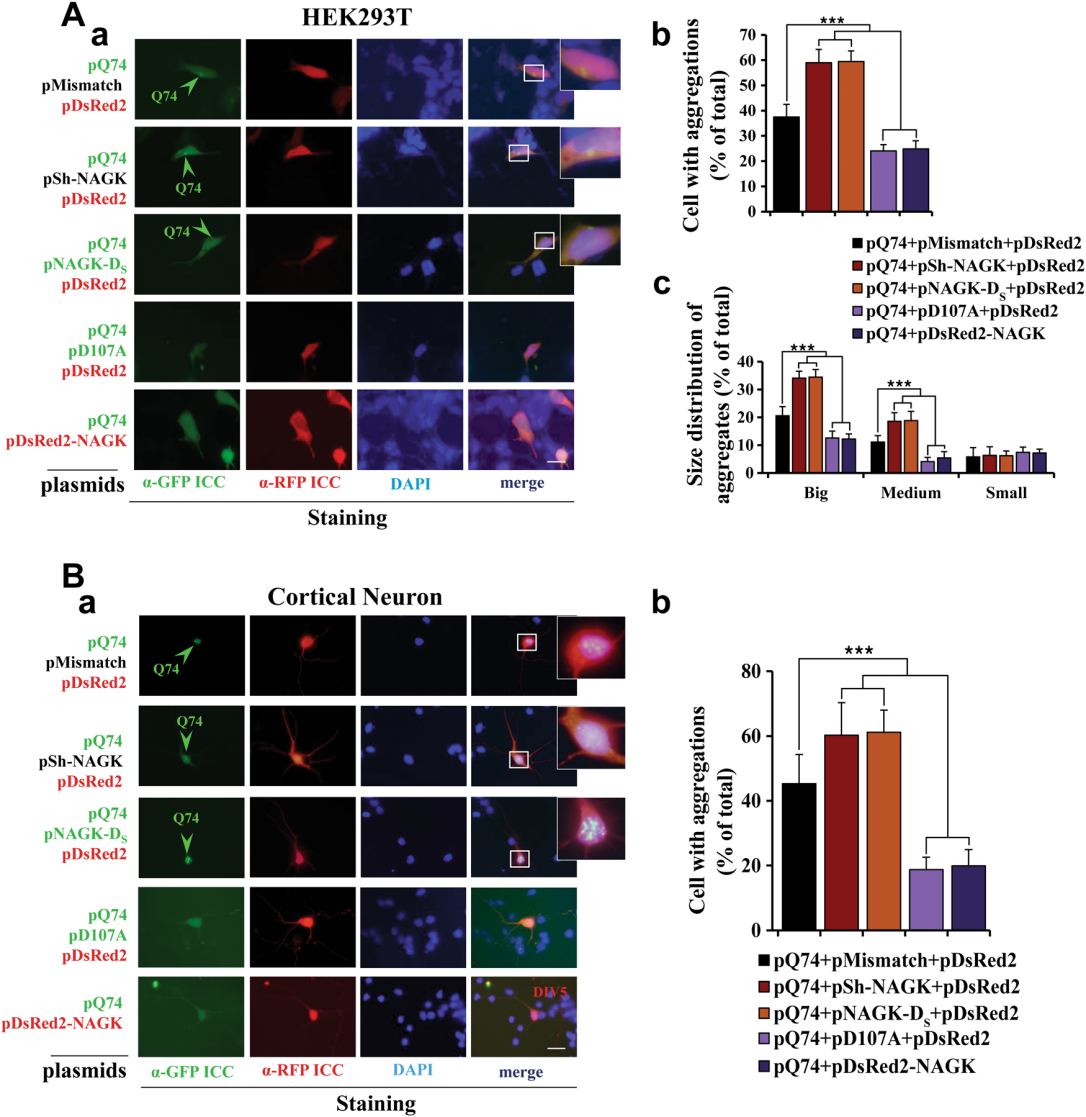
Effects of shRNA, NAGK-D_S_, and of NAGK_D107A_ on Q74 aggregate formation. **A** The HEK293T cellular model. HEK293T cells were transfected with indicated plasmids, double-labeled with indicated antibodies, and then stained with DAPI as described in Fig. 1. (a) Typical epifluorescence images. (b, c) Cells (*n* = 320) with aggregates were counted; results are expressed as percentages of all cells (b). Aggregates were classified as small (≤0.8 µm), medium (0.8–1.5 µm), or large (≥1.5 µm) and percentages in each category are shown (c). **B** The neuronal model. (a) Typical epifluorescence images. (b) Cells containing Q74 aggregates were counted; results are expressed as percentages of total cell counts (*n* = 100). Annotations are same as those used in Fig. 1. ***p < 0.001. Scale bar = 10 µm.

Next, we investigated the effects of NAGK loss-of-function on the aggregation of mHtt using a shRNA-NAGK vector (pSh-NAGK) targeting a coding region [418–438] of *Rattus norvegicus N*-acetylglucosamine kinase mRNA [NM_001037768.1], as described in our previous report^14^, and a deletion mutant of NAGK containing only the small domain (NAGK-D_S_), which had a dominant-negative effect on the non-canonical function of NAGK, that is, its effects were similar to those of sh-NAGK^14,15^. To investigate the effects of sh-NAGK and NAGK-D_S_, we triply co-transfected HEK293T cells with pQ74, pDsRed2, and a shRNA vector (pSh-NAGK) or a control mismatch vector (pMismatch). After incubation for 72 h, cells were double-immunostained with antibodies against GFP and RFP and nuclei were counterstained with DAPI. Q74 aggregates were seen in HEK293T cells transfected with pSh-NAGK (Fig. 2A–a, pQ74+pSh-NAGK + pDsRed2) or with pMismatch (Fig. 2A–a, pQ74+pMismatch+pDsRed2). HEK293T cells triple co-transfected with pQ74, pDsRed2, and pEGFP-NAGK-D_S_ (pNAGK-D_S_) also exhibited Q74 aggregation (Fig. 2A–a, pQ74+pNAGK-D_S_ + pDsRed2). Furthermore, statistical analysis showed pSh-NAGK vector and pNAGK-D_S_ significantly (*p* < 0.001) increased the percentage of aggregate-positive cells (Fig. 2A–b). The observed increase in the formation of aggregates by the dominant-negative function of NAGK-D_S_ suggested that the suppression of aggregate formation is a non-canonical function of NAGK quite distinct from its kinase activity. Encouraged by this notion, we investigated whether a kinase-inactive NAGK_D107A_ (D107A)^15^ might also suppress aggregate formation. For this purpose, we triply co-transfected HEK293T cells with pQ74, pDsRed2, and pDsRed2-NAGK_D107A_ (pD107A). After incubation for 72 h, cells were double-stained with antibodies against GFP and RFP (to reveal Q74 and DsRed2, respectively) and then DAPI stained. To our surprise, the exogenous expression of D107A suppressed the formation of Q74 aggregates (Fig. 2A–a, pQ74 + pD107A + pDsRed2). Statistical analysis showed the suppression of aggregate formation by pD107A was comparable to that observed for the wild-type NAGK version (pDsRed2-NAGK) and that the percentage of aggregate-positive cells was significantly (*p* < 0.001) lower than that in the pMismatch plus pDsRed2 co-transfected control (Fig. 2A–b).

Similar phenomena were observed in DIV 2 primary rat cortical neurons. In analogous experiments, pMismatch, pSh-NAGK, or pNAGK-D_S_ transfected neurons exhibited aggregates (Fig. 2B–a), and both pSh-NAGK and pNAGK-D_S_ significantly (*p* < 0.001) increased the percentage of aggregate-positive neurons (Fig. 2B–b) than pMismatch. Conversely, exogenous expression of DsRed2-NAGK_D107A_ (D107A) in neurons suppressed Q74 aggregation (Fig. 2B–a, pQ74 + pD107A + pDsRed2). Statistically, both D107A and wild-type NAGK (DsRed2-NAGK) very significantly (*p* < 0.001) reduced the percentage of aggregate-positive cells versus mismatch RNA or DsRed2 controls (Fig. 2B–b).

To obtain more insight, we classified aggregates by diameter as small (≤0.8 µm), medium (0.8–1.5 µm), or large (≥1.5 µm) (see Fig. 1A–a) and then performed counts in HEK293T cells (*n* = 320) transfected with pQ74 and various combinations of pDsRed2-NAGK, pD107A, pNAGK-D_S_, pMismatch, pSh-NAGK, and pDsRed2. We found co-transfection with pSh-NAGK and pNAGK-D_S_ significantly (*p* < 0.001) increased the proportions of large and medium-sized aggregates, whereas co-transfection with pDsRed2-NAGK and pD107A significantly (*p* < 0.001) reduced the proportions large and medium aggregates as compared with the pMismatch plus pDsRed2 control (Fig. 2A–c). However, differences between pDsRed2-NAGK and pD107A and between pSh-NAGK and pNAGK-D_S_ were not significant (Fig. 2A–c). Interestingly, proportions of small aggregates were no different after any co-transfection, indicating NAGK had no effect on the clearance of these small aggregates.

### Exogenous expression of NAGK erased the effects of NAGK shRNA

**Fig. 3:**
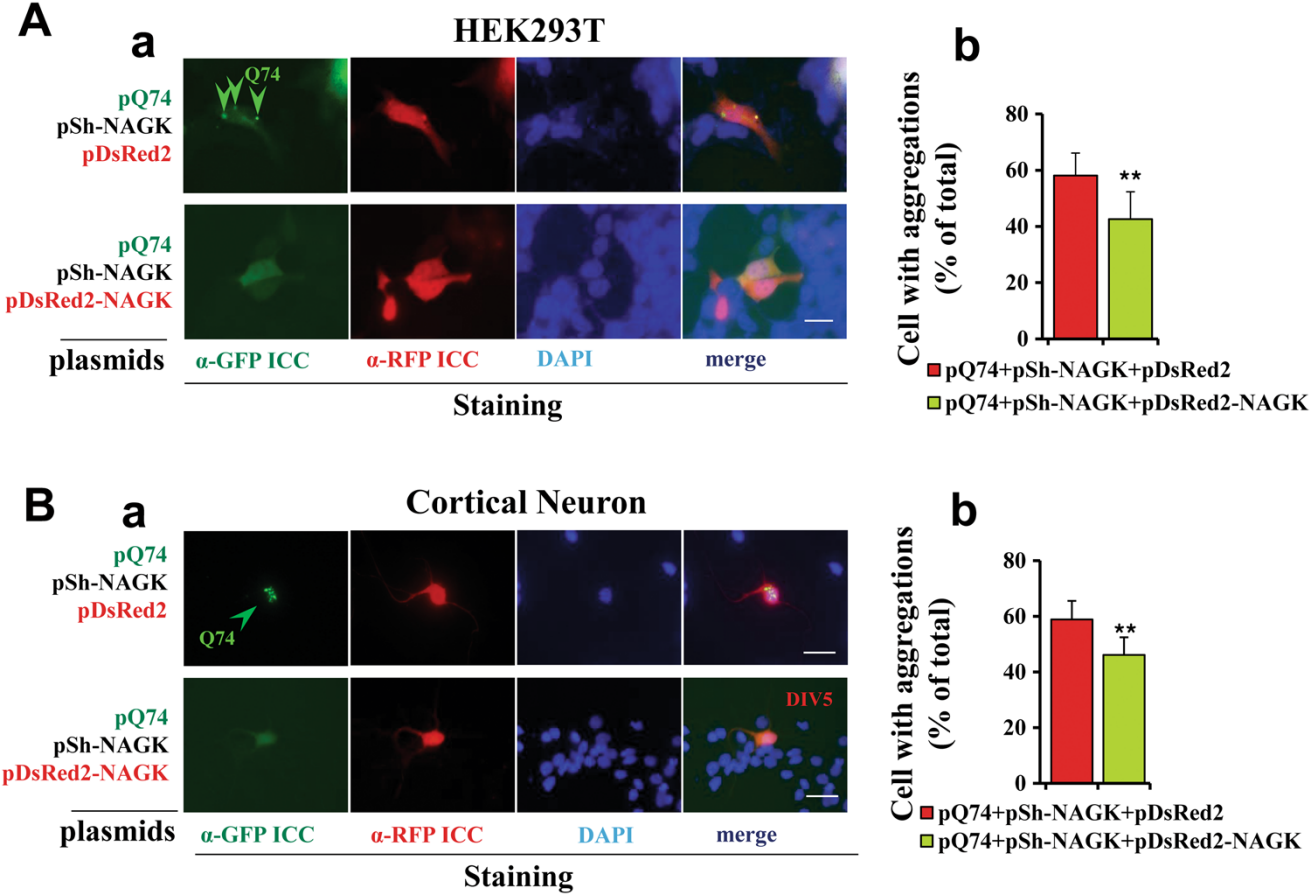
Overexpression of NAGK reversed the effects of shRNA. **A** The HEK293T cellular model. HEK293T were transfected with indicated plasmids, double-labeled with indicated antibodies (GFP and RFP), and then stained with DAPI, as described in the legend of Fig. 1. (a) Typical epifluorescence images. (b) Cells containing Q74 aggregates were counted; results are expressed as percentages of total cell counts (*n* = 300). **B** The neuronal model. (a) Typical epifluorescence images. (b) Neurons containing Q74 aggregates were counted; and results are expressed as percentages of total cell counts (*n* = 100). Annotations are the same as those used in Fig. 1. **p < 0.01. Scale bar = 10 µm.

Next, we investigated whether the aggravating effect of NAGK shRNA on aggregate clearance could be abrogated by NAGK overexpression. For this purpose, we triple co-transfected HEK293T cells with pQ74, pSh-NAGK, and pDsRed2-NAGK or pDsRed2 as a control. Immunostaining after 72 h of incubation showed that cells triple co-transfected with pQ74, pSh-NAGK, and pDsRed2 exhibited more and larger Q74 aggregates (Fig. 3A–a), whereas cells co-transfected with pDsRed2-NAGK instead of pDsRed2 showed dramatic reductions in the percentages of aggregate-positive cells (*p* < 0.01) (Fig. 3A–b). Similar phenomena were observed during analogous experiments conducted using primary rat cortical neurons. We found pDsRed2-NAGK transfection reduced Q74 aggregation (Fig. 3B–a) and significantly reduced the proportion of cells containing aggregates (*p* < 0.01) (Fig. 3B–b), indicating that the overexpression of exogenous NAGK abrogated the effect of shRNA.

### Exogenous expression of NAGK suppressed ROS generation and maintained the ‘thread-like’ morphology of mitochondria in a cellular model of HD

Mitochondrial morphology shifts from a reticular/ramified shape to a dysfunctional spherical shape in HD cellular models and patients^17^. Therefore, we investigated the effect of NAGK on mitochondrial morphology in a cellular model of HD. HEK293T cells were co-transfected with pQ74, pCMV6-Myc-DDK-NAGK (pDDK-NAGK), and pMitoTimer, the latter of which encodes a mitochondria-targeting GFP that shifts irreversibly to red fluorescence when oxidized^18^. After incubation for 24 h, cells were double-immunostained with antibodies against GFP and RFP. A series of optically dissected images of mitochondria along the *z*-axis were acquired; lower, middle, and upper layers are shown in Fig. S2A–a, which shows filamentous mitochondrial morphology resembling ‘long threads’. In contrast, mitochondrial morphologies in cells transfected with pMitoTimer and pQ74 but not pDDK-NAGK were mainly fragmented or spherical (Fig. S2A–b). Counts of numbers of mitochondria with different shapes showed the exogenous expression of DDK-NAGK significantly (*p* < 0.01) increased the proportion of filamentous mitochondria and markedly reduced (*p* < 0.001) those of fragmented and spherical mitochondria (Fig. S2A–b).

ROS overproduction leads to mitochondrial dysfunction^19^, which is a key modulator of the pathogenesis of HD, and poly (Q) mutation is known to be directly responsible for increasing ROS levels in neuronal and non-neuronal cells^4^. To observe the effect of NAGK on ROS levels, HEK293T cells were transfected with pQ74 or co-transfected with pQ74 and pDDK-NAGK. After incubation for 24 h, intercellular ROS levels were measured by treating cultures with CellROX Deep Red (Invitrogen). Co-transfected cells were found to fluoresce significantly less than pQ74 transfected cells (*p* < 0.001; Fig. S2B).

### Exogenous expression of NAGK reduced Q74 aggregation in mouse brains

**Fig. 4:**
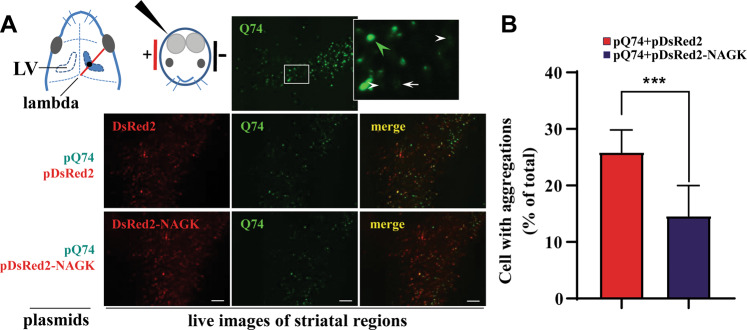
Exogenous NAGK expression suppressed Q74 aggregation in mouse brains. **A** In vivo electroporation and typical images. The indicated plasmids were injected using a glass microcapillary into the right lateral ventricles (LVs) of postnatal 1 (P1) mouse brains one-third between the right eye and lambda, as indicated by the black dot. After injection, brains were immediately electroporated by placing the anode of a tweezers-like electrode on the dorsal surface of the head to target the striatum for electroporation. Brains were sectioned at 30 μm 2 days later using a vibratome, and sections were observed under a fluorescence microscope. Typical live images of striatal regions are shown with an enlargement of the boxed area shown in the top panel, in which large (green arrowhead) and small (white arrow) Q74 aggregates are marked. White arrowheads indicate electroporated cells with no apparent aggregates. Scale bar = 50 μm. **B** Percentages of cells containing Q74 aggregations were determined in striatal regions. **p < 0.01, ***p < 0.001.

Next, an in vivo electroporation was used to study the role played by NAGK in the clearance of Q74 aggregates. Using this technique, we introduced pQ74 alone or in combination with pDsRed2 or pDsRed2-NAGK (Fig. 4) into the right ventricles of postnatal day 1 (P1) ICR mouse brains, immediately electroporated, and then returned pups to their mother. Q74-expressing green fluorescent cells were mainly observed in striatal regions at 2 days post-electroporation (Fig. 4A, top panel), and about 44% of pQ74 electroporated cells were aggregate-positive (Fig. 4B, pQ74). In contrast, the percentage of cells containing Q74 aggregates was ~25% in pQ74 + pDsRed2-NAGK electroporated brains (Fig. 4A, B), which was significantly lower than that observed in pQ74 (*p* < 0.001) and pDsRed2 (*p* < 0.01) electroporated brains (Fig. 4B). This result indicated that NAGK promoted the reduction of Q74 aggregates.

### NAGK interacted with DYNLRB1

**Fig. 5:**
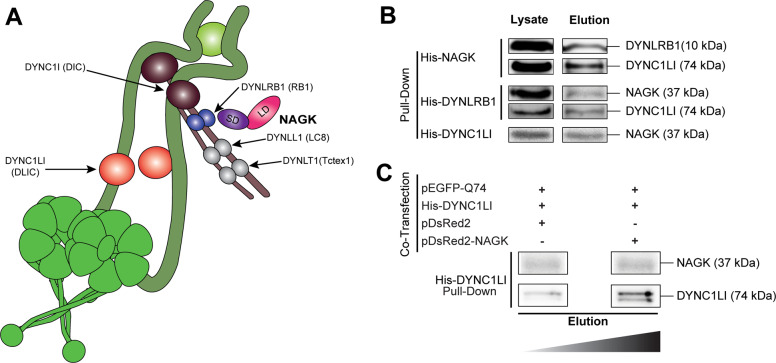
NAGK interacted with dynein by directly interacting with DYNLRB1. **A** Dynein architecture and the possible NAGK interaction site. The small domain (SD) of NAGK interacts with the dynein light chain roadblock 1 (DYNLRB1). **B**, **C** Co-immunoprecipitation. Exogenous His-tagged NAGK, His-tagged DYNLRB1, and His-tagged DYNC1LI were expressed in HEK293T cells, pulled-down using Ni-NTA magnetic beads, and immunoblotted with indicated antibodies (**B**). The interaction between DYNC1LI and NAGK was greater in the HD cellular model. His-tagged DYNC1LI was co-transfected with pQ74 and pDsRed2 (Control) or pDsRed2-NAGK and incubated for 48 h (**C**).

In a previous report, we showed that NAGK interacts with DYNLRB1 in cytoplasmic dynein^11^ (Fig. 5A), and suggested these interactions might be involved in the protein trafficking of Q74 aggregates. To examine this interaction, His-tagged NAGK plasmid was transfected into HEK293T cells, and then dynein complex was pulled down. Exogenous His-tagged NAGK was found to associate with endogenous DYNLRB1 and light intermediate chain 1 (DYNC1L1), as detected by immunoblotting with respective antibodies (Fig. 5B). Similarly, NAGK was also detected by immunoblot by pulling down dynein expressed exogenously by His-tagged DYNLRB1 or His-tagged DYNC1L1, which confirmed NAGK interacts with dynein at the basal level.

Next, we examined whether the effect of applying misfolding stress to cells on NAGK-dynein complex. For this purpose, we used an HD cellular model, in which pQ74 and His-tagged DYNC1L1 were co-transfected in combination with pDsRed2 or pDsRed2-NAGK in HEK293T cells, and dynein complex was pulled down using Ni-NTA magnetic beads. NAGK-dynein interaction was observed in this model, and the association between NAGK and dynein was found to be greater in pDsRed2-NAGK transfected cells than in pDsRed2 transfected cells (Fig. 5C). This observation showed that the NAGK-DYNLRB1 interaction facilitates the dynein motor protein trafficking of aggregated proteins.

### Structural modeling of the DYNLRB1-NAGK interaction

**Fig. 6:**
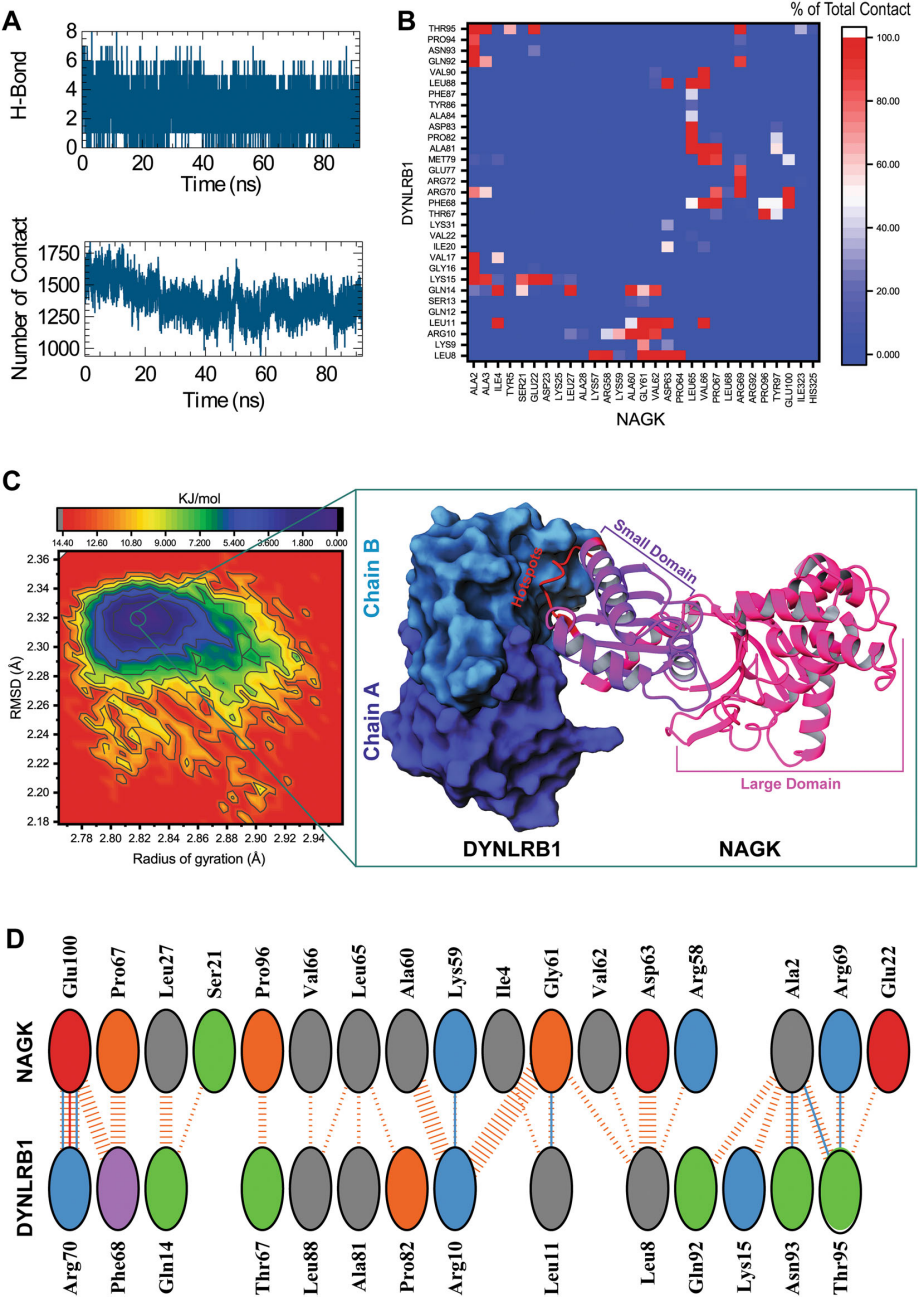
Molecular modeling of NAGK-DYNLRB1 complex revealed the critical NAGK binding site and the nature of the interaction between NAGK and DYNLRB1. **A** The intermolecular interaction patterns obtained by MDS provided the total number of hydrogen bonds (upper panel) and contacts (lower panel) formed during a simulation time of 92 ns. **B** Intermolecular contact percentages summarized in the heatmap represent magnitudes of inter-residue contacts over the simulation period. Here, the red to blue color-coded bar represents higher to lower total contact percentages. **C** Free energy landscape analysis provided insight of the conformational dynamics of NAGK-DYNLRB1 complex. The dark blue regions denote the distribution of the conformer with the lowest energy minimum (0 kJ/mol). **D** 2D intermolecular interaction plot of the most stable conformer obtained by free energy landscape analysis, which was available at a corresponding radius of gyration (Rg) and RMSD to the average value of 2.82 and 2.31 nm, respectively. The plot highlights major nonbonded interactions at the NAGK/DYNLRB1 protein–protein interface.

Next, we investigated the NAGK-DYNLRB1 interaction by in silico molecular modeling. First, we performed protein–protein docking based on considerations of the dimeric form of DYNLRB1 (chains A and B) with NAGK, because an earlier report suggested DYNLRB1 exists as a dimer in dynein complex^20^. The NAGK-DYNLRB1 complex used from molecular docking analysis was selected using binding energies calculated using the FoldX program, accordingly to which the binding energy of the most favorable dimer was −4.02 kcal/mol. NAGK is composed of small (residues 1–117) and large (residues 118–344) domains, and the small domain interacts with the C-terminal DYNLRB1, as we previously reported^11^. Consistently, the resulting NAGK-DYNLRB1 complex exhibited similar orientations of NAGK to DYNLRB1, and thus, it was subjected to molecular dynamics simulation (MDS). Simulation stability was checked using root-mean-square deviations (RMSDs) (Fig. S3A), which showed that NAGK and DYNLRB1 in the complex achieved equilibration after 15 ns and then remained stable. For DYNLRB1, the RMSD curve of chain B was more stable than that of chain A, which indicated NAGK interacted more strongly with chain B during the simulation. Therefore, we measured total contact and hydrogen bonding between DYNLRB1 chain B and NAGK (Fig. 6A) after 25 ns of simulation when interactions remained stable. A heatmap based on total contact percentage showed that residues, Lys57 to Arg69 of NAGK small domain made greatest contact with DYNLRB1 (residues, Ala81 to Val90 and Leu8 to Leu11) throughout the simulation (Fig. 6B). For more insight, we performed free energy landscape (FEL) analysis and extracted the most energetically favorable conformation (0 kJ/mol) at 67.52 ns (Fig. 6C). The 2D interaction map of the retrieved complex showed that residues Lys57 to Arg69 of NAGK participated in hydrophobic and electrostatic interactions with the C-terminal of DYNLRB1, in which, Lys59, Gly61, and Arg69 participated in hydrogen bonding interactions. In addition, Gly61 also interacted hydrophobically with Arg10 and Leu8 of DYNLRB1 (Fig. 6D).

### NAGK derived peptide interfered with Q74 clearance

**Fig. 7:**
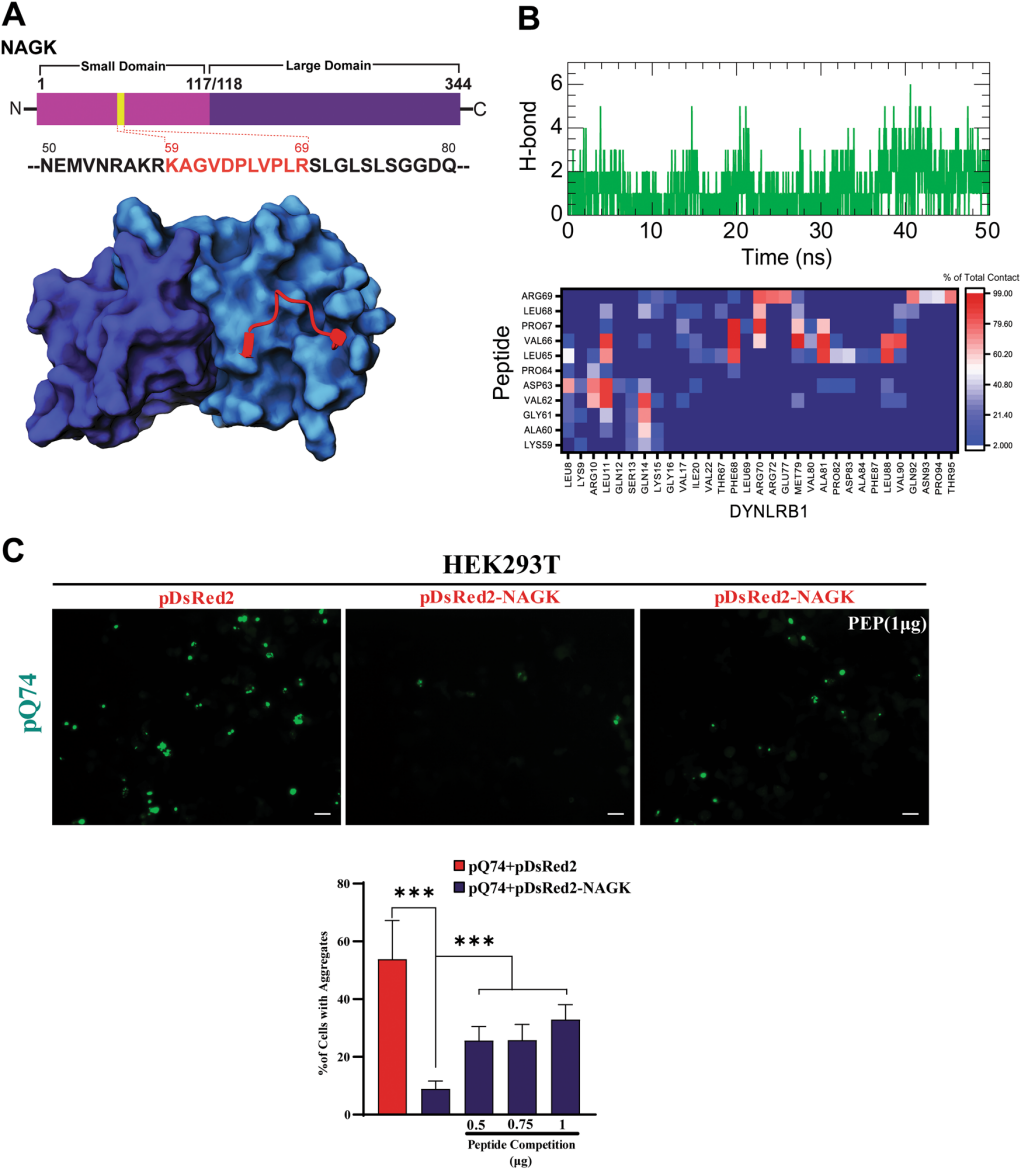
DYNLRB1 specific NAGK peptide inhibited NAGK-mediated Q74 clearance. **A** Design of NAGK peptide based on the modeled NAGK-DYNLRB1 interaction interface. The upper left panel shows peptide mapping (K^59^AGVDPLVPLR^69^) of the NAGK small domain, and the panel at the lower left shows the interaction between DYNLRB1 and peptide. **B** Time-course changes in numbers of intermolecular contacts during MDS. The right upper panel shows the number of hydrogen bonds formed between NAGK peptide and DYNLRB1 in a 50 ns MDS, while the right lower panel shows a heatmap representing magnitudes of inter-residue contact formation between NAGK peptide and DYNLRB1 over the simulation. **C** Transfection of NAGK peptide inhibited the NAGK-mediated suppression of Q74 aggregates in the cellular HD model (HEK293T). HEK293T cells were initially co-transfected with pQ74 and pDsRed2 or pDsRed2-NAGK (representative immunofluorescence images are shown in the left and middle panels). The HEK293T cells initially co-transfected with pQ74 and pDsRed2-NAGK were then re-transfected 6 h later with the NAGK-D_S_ derived peptide (K^59^AGVDPLVPLR^69^, right panel), and further incubated for 48 h (right panel). Green puncta represent Q74 aggregates. The lower panel represents statistics and shows transfection with the peptide significantly increased the proportion of cells containing aggregates. ***p < 0.0001, *n* = 100, scale bar = 20 µm.

MDS showed that the small domain of NAGK (region, K^59^AGVDPLVPLR^69^) binds specifically to the C-terminal DYNLRB1 (Fig. 7A), and thus, we designed a peptide to target this region to confirm the location of the NAGK binding site in DYNLRB1. A peptide-DYNLRB1 complex was designed from a FEL-derived conformer, and its binding was simulated for 50 ns to determine binding feasibility. During the simulation, the peptide frequently maintained H-bonding to DYNLRB1, and its interaction was greatest during the last 10 ns of simulation (Fig. 7B, upper panel). The heatmap showed that the peptide made significant contact with DYNLRB1 (Fig. 7B, lower panel), notably, with Gly61, Val62, Asp63, Leu65, Val66, Pro67, Leu68, and Arg68, which indicated strong binding.

We then examined whether peptide binding to DYNLRB1 influences the NAGK-DYNLRB1 interaction and affects aggregate clearance. For this purpose, we first co-transfected HEK293T cells with pQ74 and pDsRed2 or pDsRed2-NAGK, and 6 h later, transfected cells with different concentrations of the peptide. Remarkably, pDsRed2-NAGK transfection significantly (*p* < 0.0001) reduced the percentage of aggregate-positive cells as compared with pDsRed2 transfected control cells, which reconfirmed our earlier results (Fig. 7C). Moreover, the peptide significantly and concentration-dependently increased the percentage of aggregate-positive cells (*p* < 0.0001) as compared with pDsRed2-NAGK control cells. Collectively, these observations suggested that the designed peptide specifically inhibited the NAGK to DYNLRB1 interaction and inhibited Q74 clearance.

## Discussion

**Fig. 8:**
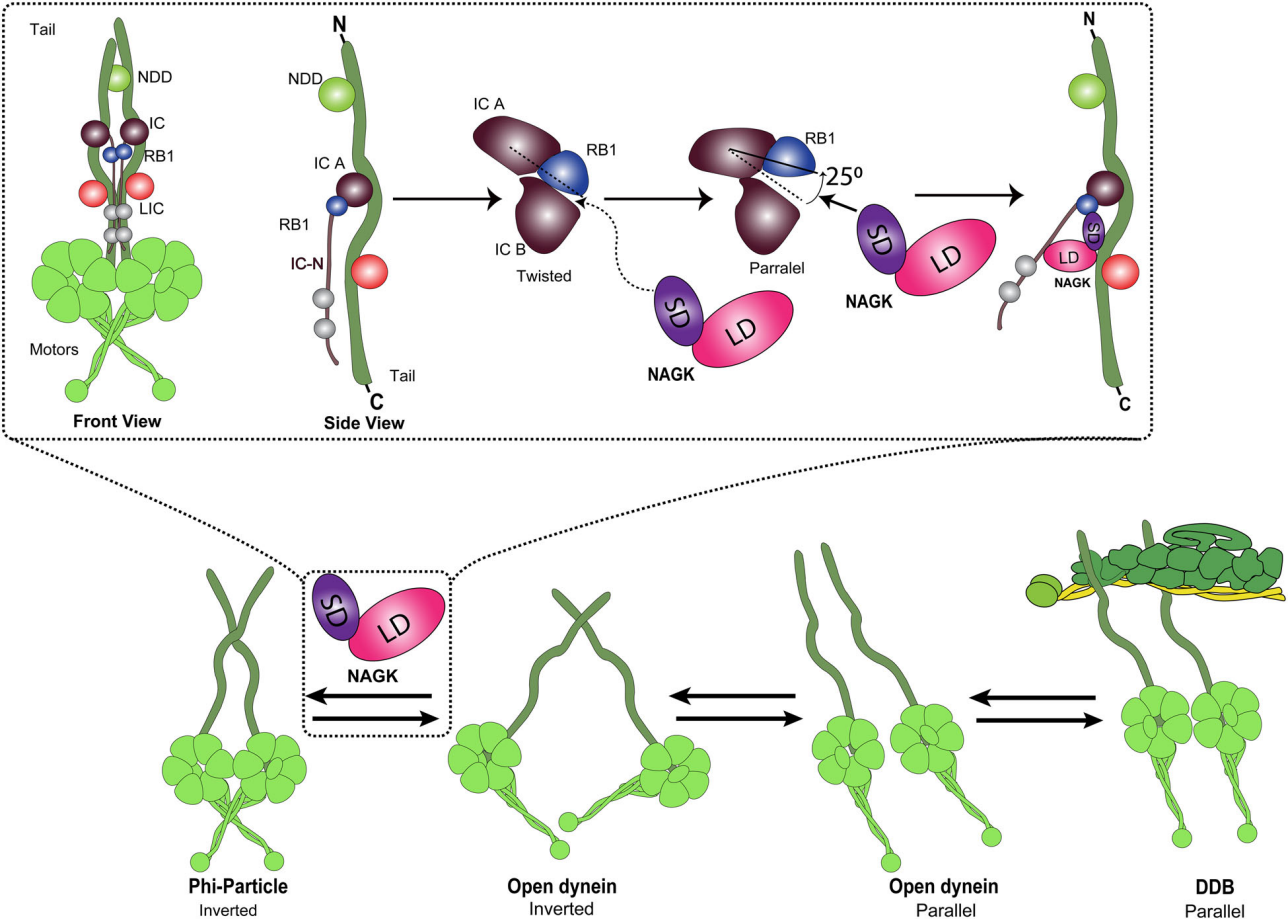
A proposed model for dynein activation by NAGK-DYNLRB1 (RB1) interaction. The activation of dynein is accomplished by a structural transition from phi- to open-dynein, where LCs trail (dark brown) away from the HC tail (dark green)^33^. In the phi conformation, dynein exists in a ‘twisted’ state, whereas in a ‘parallel’ state it can bind to dynactin, and hence, is activated. During the ‘twisted’ to ‘parallel’ transition, RB1 rotates by 25°, which induces a large positional change of the LC8/Tctex tail causing it to move away from the HC neck. We propose binding of NAGK to RB1 causes the RB1-IC A to rotate. The resulting shift in the relative positions of the two ICs (IC A and IC B) confers momentum for the phi- to open-dynein transition and ‘pushes up’ the LC tail. IC-N, N-terminal of intermediate chain; LD and SD, large and small domain of NAGK; NDD, N-terminal dimerization domain; DYNLRB1 (RB1), dynein light chain roadblock 1.

When chaperone and ubiquitin-proteasome pathways are disrupted or overloaded, protein aggregates are transported by cytoplasmic dynein to cell centers, where aggresomes are formed and degraded by autophagy^21^. However, defects in autophagy regulation have been suggested in many NDs, and thus, autophagy activation has been proposed as a potential means of clearing abnormal protein aggregates^22–25^. In this study, we tested the hypothesis that promoting the function of dynein would result in the clearance of protein aggregates. In neuronal and non-neuronal cellular models of HD and PD, exogenous NAGK expression reduced mutant huntingtin and α-synuclein aggregates, and this was attributed to a non-canonical effect of NAGK, and was associated with reduced ROS levels and the presence of mitochondria with a healthy ‘thread-like’ morphology. The reduction of mHtt aggregates by NAGK was further verified by in vivo electroporation of P1 mouse brains. Furthermore, inhibition of the DYNLRB1-NAGK interaction by a specific peptide derived from the small domain of NAGK inhibited aggregate clearance in a cellular HD model. Results are summarized in Fig. 8.

We found NAGK in DsRed2- or DDK-tagged forms efficiently reduced Q74 and α-syn A53T aggregates in cultured neuronal and non-neuronal cells and Q74 aggregates in mouse brains, which suggested NAGK either suppressed aggregate formation or promoted their removal. We favor the latter scenario for two reasons. First, NAGK promotes the function of cytoplasmic dynein^11–15^. If aggregates were disposed of via the ALP, efficient transport of aggregates to MTOC by dynein-NAGK complex would facilitate formation of aggresome, autophagosome, and autolysosome, because lysosomes are highly concentrated at the cell center (i.e., MTOC)^7^, accelerated transport would aid aggregate clearance. Second, NAGK expression did not affect the number of small aggregates, whereas large and medium aggregates were significantly fewer in NAGK-overexpressing cells. Q74-expressing cells always exhibited green fluorescence, and small aggregates were consistently observed in cytoplasm. Although the threshold size of aggregates required for dynein transport is not known, our results indicate that an aggregate must attain a certain size before it is disposed of via the ALP. This suggestion regarding minimal aggregate size is supported by the gathering of scattered polyubiquitinated protein aggregates induced by HDAC6, which plays an essential role in transporting protein aggregates via microtubule tracts^26^, and thus, favors aggresome formation and autophagy^27^. HDAC6 binds to both polyubiquitinated aggregates and dynactin/p150Glued (a component of dynein motor complex), and links ubiquitinated proteins to dynein motor and promotes cargo transport to MTOC^28^. Thus, we believe the background green fluorescence observed in Q74-expressing cells was caused by small scattered aggregates.

The present study shows that protein aggregate reduction by exogenous NAGK suppressed ROS generation and helped maintain a ‘thread-like’ mitochondrial morphology in our cellular model of HD. Extensive evidence shows dysregulation of autophagy increases oxidative stress, as has been demonstrated by pharmacological inhibitor and knockout studies^29,30^, and that oxidative stress can lead to non-specific post-translational modifications of proteins and contribute to protein aggregation. Hence, it is likely that protein aggregate clearance would reduce ROS levels and support mitochondrial health. Indeed, the present study shows NAGK overexpression significantly reduced numbers of dysfunctional spherical mitochondria and increased numbers of ‘thread-like’ healthy mitochondria in our HD cellular model, which suggests NAGK promoted ALP activity by dynein activation and consequently reduced ROS levels and mitochondrial damage.

The present study also shows that aggregate reduction is a non-canonical effect of NAGK. Previously, we reported NAGK is highly expressed in neurons, and that the exogenous expression of NAGK upregulated the formation of dendrites^14^. This upregulation was found to be a non-canonical function of NAGK, because enzyme activity-nullified D107A mutant NAGK also upregulated dendritic arborization as effectively as the wild-type^15^. Furthermore, exogenous expression of the small domain of NAGK (NAGK-D_S_) resulted in dendrite degeneration^15^, indicating NAGK-D_S_ played a negative-dominant effect by a structural role in dynein regulation. Indeed, the ectopic introduction of a small peptide derived from the C-terminal amino acids 74–96 of DYNLRB1 resulted in the stunting of hippocampal neuron axons^11,13^. In the present study, overexpression of NAGK-D_S_ abrogated aggregate clearance by NAGK and aggravated aggregation, and the NAGK-D_S_-derived peptide K^59^AGVDPLVPLR^69^ nullified the effect of wild-type NAGK on Q74 clearance. These results all support that NAGK plays a structural role in dynein activation.

Then how could NAGK activate dynein? The activation of dynein is accompanied by a structural change from the phi to the open form, and previous negative stain EM studies on open-dynein showed its light chains (LCs) trail from the heavy chain (HC) tail^31^. The phi- to open-dynein transition is also accompanied a conformation change of the 200-amino-acid N-terminal dimerization domain of the HC tail from a ‘twisted’ to a ‘parallel’ state. When dynein is bound to dynactin, the conformation of this region most resembles the parallel state^32^. Interestingly, the twisted to parallel transition correlates with small shifts in the relative position of the two ICs (IC A and IC B) and larger changes in the position of the LCs (DYNLRB1, LC8/Tctex). Specifically, a small shift in the relative position of the IC A and IC B causes DYNLRB1 rotates through 25° and the LC8/Tctex density moves away from the HC neck^33^. We reason that NAGK binding to DYNLRB1 causes a shift in the relative position of the two ICs. To be specific, DYNLRB1 is composed of a 10-stranded β-sheet core, which provides a positively charged surface area with major protein–protein interaction sites for its partner, IC, through residues 79–82, 88, and 90^20^. Interestingly, this interaction domain of DYNLRB1 is also the NAGK binding site, as indicated by the present study and our previous report^11^. As shown by our molecular modeling studies, residues 59–69 in the small domain of NAGK are the major hotspots for DYNLRB1 binding. Thus, there seems to be a binding competition between NAGK and IC for DYNLRB1. If NAGK wins, it will bind DYNLRB1, causing a shift in the relative position of the two ICs and DYNLRB1 rotation, resulting in a phi- to open-dynein structural transition and a ‘push up’ event of the LC tails (Fig. 8). A cryo-EM study is needed to confirm this proposition.

In summary, we report NAGK acted in a non-canonical manner to reduce protein aggregates in neuronal and non-neuronal cellular models of HD and PD, and that these suppressions of protein aggregates also prevented mitochondrial damage and reduced ROS production. At the molecular level, the small domain of NAGK interacted with the C-terminal DYNLRB1, which may have activated dynein motor.

## Materials and methods

### Antibodies and plasmids

The following antibodies were used at the indicated dilutions: rabbit polyclonal antibody against RFP (DsRed2; 1:1000, Chemicon, Temecula, CA, USA) and against DYNLRB1 (1:1000, ABclonal, MA, USA); mouse monoclonal antibodies against GFP (12A6; 1:25, Developmental Studies Hybridoma Bank, University of Iowa, Iowa City, IA, USA), against α-syn (1:50; 211; Santa Cruz Biotechnology, Dallas, Texas, USA), against NAGK (1:1000; Santa Cruz Biotechnology, Texas, USA) and DYNC1LI1 (1:1000, Millipore Sigma, Burlington, MA, USA). Secondary antibodies (Alexa Fluor 488-conjugated goat anti-mouse IgG and Alexa Flour 568-conjugated goat anti-rabbit IgG) were from Invitrogen (Carlsbad, CA, USA). pEGFP-Q74 (Q74; this plasmid has a HTT partial exon 1 Q74 insert)^34^ pEGFP-Q74 was a gift from David Rubinsztein (Addgene plasmid # 40262; http://n2t.net/addgene:40262) and pHM6-alphasynuclein-A53T was a gift from David Rubinsztein (Addgene plasmid # 40825; http://n2t.net/addgene:40825)^35^. pMitoTimer was a gift from Zhen Yan (Addgene plasmid # 52659; http://n2t.net/addgene:52659)^18^. pDsRed2 vector was purchased from ClonTech (now Takara Bio USA, Inc., Mountain View, CA, USA) and pCMV6-Myc-DDK-tagged rat NAGK (pDDK-NAGK) was from OriGene Technologies, Inc. (RR206612; Rockville, MD, USA). pDsRed2-tagged wild-type NAGK (pDsRed2-NAGK), pEGFP-tagged point mutant NAGK (pEGFP-NAGK_D107A_), deletion mutant containing only the small domain of NAGK (pEGFP-NAGK-D_S_), NAGK short hairpin (pSh-NAGK), and mismatch RNA vectors (pMismatch) were previously described^14,15^. His-tagged NAGK (pENTER-CMV-NAGK), DYNC1LI1 (pENTER-CMV-DYNC1LI1), DYNLRB1 (pENTER-CMV-DYNLRB1) were purchased from Vigene Biosciences (Rockville, MD, USA).

### Cell culture and transfection

Cortical cells from embryonic day 19 (E19) Sprague-Dawley rat brains (Orient Bio Inc., Seongnam-si, Korea) were cultured as previously described^36^. Experiments were approved beforehand by the Institutional Animal Care and Use Committee of the College of Medicine, Dongguk University (Approval no: IUCAC-2018-06). Cells were initially plated in MACS^®^Neuro Medium (Miltenyi Biotec Inc., USA) supplemented with MACS NeuroBrew^®^-21, 45.95 µM glutamate, 500 µM glutamine, 25 µM 2-mercaptoethanol, and 1% penicillin-streptomycin, and fed every four days with the same medium without glutamate or 2-mercaptoethanol supplementation. HEK293T cells were cultured on polylysine-coated glass coverslips in DMEM (Invitrogen) containing 10% fetal bovine serum and 1% penicillin-streptomycin. Cells were transfected using Lipofectamine^®^ 2000 reagent (Invitrogen), and immunostained after fixing with paraformaldehyde and methanol^37^.

### Measurement of intracellular ROS levels

Transfected HEK293T cells were incubated with 5 µM CellROX Deep Red (Invitrogen) for 30 min at 37° C in a 5% CO_2_ atmosphere and washed twice with PBS. Fluorescent images were acquired using an Olympus BX53 microscope (Olympus, Tokyo, Japan). Mean fluorescence intensities were measured using Image J software (version 1.45, National Institute of Health, Bethesda, MD).

### In vivo electroporation of neonatal mice and imaging

Electroporation was essentially performed as described by Ito et al.^38^. In brief, postnatal day 1 (P1) ICR mice were anesthetized by hypothermia, and then skin and skull were pierced with a sharp glass microcapillary containing a solution of plasmid [pEGFP-Q74 (3 μg/μl), pEGFP-Q74 (3 μg/μl) + pDsRed2 (2 μg/μl), or pEGFP-Q74 (3 μg/μl) + pDsRed2-NAGK (2 μg/μl)] in Tris-EDTA buffer containing 0.5% Fast Green. DNA solution (2 µl) was injected into lateral ventricles (LVs), and injected brains were immediately electroporated with a tweezers-type electrode using an ECM 830 electroporator (BTX, USA). Five pulses of 100 V of 50 ms duration were administered with a 950 ms pause between pulses. Electroporated animals were placed on a hot pad at 37 °C for several minutes and then returned to their mother. Pups were anesthetized by hypothermia 2 days later, and brains were isolated, fixed for 2 h at RT in 4% (w/v) PFA, washed in PBS, and incorporated into agarose (5%) blocks, which were then sectioned (30 μm) with a vibratome (DTK-2000 Microslicer, Dosaka EM Co., Kyoto, Japan). Sections were mounted on slides and images were acquired using a Leica DM IRE2 Research Microscope (Leica Microsystems AG, Wetzlar, Germany). Experiments were approved beforehand by the Institutional Animal Care and Use Committee of the College of Medicine, Dongguk University.

### Ni-NTA-based pulldown of His-tagged protein complexes and immunoblotting

HEK293T cells were cultured in DMEM (Invitrogen) containing 10% fetal bovine serum and 1% penicillin-streptomycin. Cells were transfected with His-tagged plasmids (pENTER-CMV-NAGK, pENTER-CMV-DYNC1LI1, pENTER-CMV-DYNLRB1) using Lipofectamine^®^ 2000 reagent (Invitrogen). The transfection efficiencies of all plasmids ranged from 70 to 80%. After incubation for 48 h, cells were washed with cold PBS and lysed in lysis buffer (25 mM Tris, 150 mM NaCl, 1.0 mM EDTA, 1% NP-40, 5% glycerol; pH 7.4) containing protease inhibitor cocktail (Thermo Scientific, Rockford, IL). The supernatant was allowed to bind with MagListo™ Ni-NTA magnetic silica resin (Bioneer, Daejeon, Korea) and pulled down according to the manufacturer’s instructions. After washing four times with binding buffer, His-tagged protein was eluted using elution buffer (0.5 mM imidazole, pH 8.0). The eluted proteins were separated by SDS-PAGE (15% Tricine-SDS-PAGE in the case of DYNLRB1) gel and transferred to PVDF membranes, which were incubated with primary antibodies: anti-NAGK (1:1000; mouse monoclonal, Santa Cruz Biotechnology, Texas, USA), anti-DYNC1LI1 (1:1000; mouse monoclonal, Millipore Sigma, Burlington, MA), or anti-DYNLRB1 (1:1000, rabbit polyclonal, ABclonal, MA, USA). After rinsing with TTBS (0.05% Tween-20 in TBS), membranes were incubated with horse radish peroxidase-conjugated secondary antibodies (1:1000; anti-mouse or -rabbit IgG; Amersham Biosciences, now GE Healthcare Life Sciences, USA), and blots were detected using an ECL detection kit (Invitrogen, Waltham, MA). In some experiments, a stripping buffer was used to strip membranes (Pierce Biotechnology, Rockford, IL), and hybridization was performed with different primary and secondary antibodies.

### Protein–protein docking and structural modeling

Molecular modeling of DYNLRB1-NAGK complex was performed by protein–protein docking simulation using the SwarmDock (https://bmm.crick.ac.uk/svc-bmm-swarmdock/) server^39^, which uses the SwarmDock algorithm to predict interactions between two proteins while maintaining flexibility. Before docking, the three-dimensional structures of NAGK and DYNLRB1 were downloaded from the protein databank and prepared for docking simulation by adding hydrogens, adjusting bond orders and charges, and deleting water molecules using the protein preparation wizard of Schrödinger 2017-1 (Schrödinger, LLC, New York, NY, 2017). All structures were optimized at neutral pH and then some thiol and hydroxyl groups, amide groups of asparagines, glutamines, and the imidazole rings of histidines, protonation states of histidines, aspartic acids and glutamic acids were readjusted. Force field OPLS_2005, minimization was then performed with maximum heavy atom RMSD set at 0.30 Å. Resultant docked complexes were further subjected to binding energy calculations using the FoldX^40^ program, which uses an empirical force field algorithm to calculate ΔG in kcal/mol and considers energy function terms including van der Waals, solvation, H-bond, electrostatic and entropic terms for the backbones and side chains of proteins and protein complexes.

The complex with lowest binding energy was subjected to MDS using YASARA dynamic software (YASARA Biosciences GmBH, Vienna, Austria), as previously described^41^. The structure was cleaned, the hydrogen bond network was optimized, and a cubic simulation cell was built in accord with the periodic boundary condition. For the simulation, we applied the AMBER14 force field. To solvate the simulation cell, we used the transferable intermolecular potential3 points (TIP3P) solvation system at a density of 0.997 g/L and determined acid dissociation constant values (pKa). To maintain physiological conditions, the pH of the system was set at 7.4. Protonation states of all amino acids were calculated using a combination of the H-bonding network and the SCWRL algorithm^42^. Na^+^ and Cl^−^ ions were added to neutralize the system, and a simulated annealing protocol was used to reduce system conformational stress. The Ewald particle mesh (PME) was applied to detect long-range electrostatic interactions using 8 Å as distance cut-off. Molecular dynamics simulation was conducted for 92 ns and the Berendsen thermostat was then applied at a time-step interval of 2.00 fs, using multiple time-step algorithms^43^. The pressure was kept constant, and trajectories were saved at 10-ps intervals. All simulation phases were run using a preinstalled macro (md_run.mcr) in YASARA suite, and results were analyzed using YASARA and VMD (Version 1.9.3, Theoretical and Computational Biophysics Group, Urbana, IL, USA, 2016)^44^ tools. Finally, the most stable simulation structure was retrieved by free energy landscape (FEL) analysis^41,45^. Protein stability was assessed using Gibb’s free energy, calculated as follows:

$$G_{\mathrm{i}} = - K_{\mathrm{B}}T{\mathrm{ln}}\left( {N_{\mathrm{i}}/N_{{\mathrm{max}}}} \right)$$

Where, *K*_B_ is Boltzmann’s constant, *T* is temperature (300 K), *N*_i_ and *N*_max_ are population in bin I and the most inhabited population bin, respectively. A color code model (in Fig. 6C) depicts different energy levels.

### Peptide derived mHtt clearance assays

The NAGK peptide (K^59^AGVDPLVPLR^69^) from its small domain was custom made by GL Biochem Ltd. (Shanghai, China). The binding interaction between NAGK peptide and DYNLRB1 was analyzed over 50 ns by MDS, and this was followed with a protocol similar to that described above for DYNLRB1-NAGK complex. For inhibition assays, HEK293T cells were seeded in 24-well plates and maintained in high-glucose Dulbecco’s modified Eagle medium (DMEM; Life Technologies) supplemented with 10% fetal bovine serum (Life Technologies) and incubated for 24 h at 37 °C and in a 5% CO_2_/95% air atmosphere. Cells were then transiently co-transfected with pEGFP-Q74^34^ and pDsRed2 or pDsRed2-NAGK plasmids using the Lipofectamine 2000 (Invitrogen) transfection kit. After 6 h of transfection, 0.5, 0.75, or 1.0 μg of NAGK peptide was co-transfected with Alexa Fluor 647-conjugated goat anti-mouse IgG using Pierce protein transfection reagent (Thermo Fisher, USA). Briefly, 2.5 µL of Pierce Reagent was mixed with 0.5, 0.75, or 1.0 μg of NAGK peptide and 1.0 μl of antibody in 25 μl of PBS, incubated for 5 min, and then mixed with 250 µl of serum-free medium. Cells were then incubated in this serum-free medium containing complex and incubated for ~48 h with subsequent feeding medium, and observed under an epifluorescence microscope (Leica Research Microscope DM IRE2; Leica Microsystems AG, Wetzlar, Germany)^37^.

### Statistics

Numbers of aggregates, shapes of mitochondria, and ROS levels were determined using three independent experiments (and results are presented as means ± SDs). Counting was performed in a blinded way by two experienced independent investigators. Animals were taken from random cages within each cohort. Sample sizes were chosen based on considerations of data variability, but were never <5. The Student’s *t*-test was used to compare two groups, and one-way ANOVA with Duncan’s multiple comparison post hoc test was used for multi-group comparisons. Tests for normality were performed to justify the use of parametric tests. *P* values of <0.05 were deemed statistically significant, and *P* values of <0.0001 were considered highly significant. Statistical analysis was performed using SPSS 19.0 (SPSS Inc., Chicago, IL, USA), and GraphPad Prism v 8.0 (GraphPad Software, San Diego, USA).

## Supplementary information

Supplementary Figure Legends

Figure S1.

Figure S2.

Figure S3.
