# Supplementary material for: N-acetyl-D-glucosamine kinase binds dynein light chain roadblock 1 and promotes protein aggregate clearance
Source: Cell Death Dis. 2020 Aug 14;11(8):619. doi: 10.1038/s41419-020-02862-7 (PMC7427805; doi:10.1038/s41419-020-02862-7)
Supplement: Supplementary file 1 — Supplementary Figure Legends [file 41419_2020_2862_MOESM1_ESM.docx]

**Supplemental figure legends**

**Fig. S1. Exogenous expression of NAGK suppressed the formation of α-synuclein (α-syn) aggregates in a cellular model of PD.**

Experiments analogous to those described in the legend of Fig. 1 were carried out with pHM6-alphasynuclein-A53T (pα-syn A53T) plasmid rather than pQ74 plasmid. Annotations are same as used in Fig. 1. ***p <* 0.01, n=100. Scale bar = 10 µm.

**Fig. S2. Exogenous NAGK expression reduced ROS and maintained the healthy morphology of mitochondria in the cellular model of HD.**

HEK293T cells were co-transfected with pQ74, pDDK-NAGK and pMitoTimer. **(**A) Analysis of mitochondrial morphology. (a) Typical epifluorescence images. A series of z-stack live images were acquired 24 hr after transfection and bottom, middle, and top images are shown. Scale bar = 10 µm. (b) Cells (n=177) with filamentous ('thread-like'), fragmented (spherical), and mixed mitochondria were counted, and percentages of cells in each category are shown. (B) Analysis of ROS levels. HEK293T cells were transfected with pQ74 alone or co-transfected with pQ74 and pDDK-NAGK and 24 hr later transfected cells were stained with vibrant CellROX Deep Red dye (Invitrogen). Fluorescence intensities are expressed as means ± SDs (n=300). Statistical significance was determined using the Student's *t*-test. ***p* < 0.01, ****p* < 0.001.

**Fig. S3.** **Protein-protein docking and molecular dynamics simulation.** (A) Protein-Protein docking score and binding energy evolution for NAGK-DYNLRB1 complex. (B) Protein thermodynamics stability during the simulation was evaluated through root-mean-square deviations (RMSDs) for NAGK-DYNLRB1 complex, by considering backbone atoms (C, Cα, and N) of protein.
