# Supplementary figures and images for: N-acetyl-D-glucosamine kinase binds dynein light chain roadblock 1 and promotes protein aggregate clearance
Source: Cell Death Dis. 2020 Aug 14;11(8):619. doi: 10.1038/s41419-020-02862-7 (PMC7427805; doi:10.1038/s41419-020-02862-7)

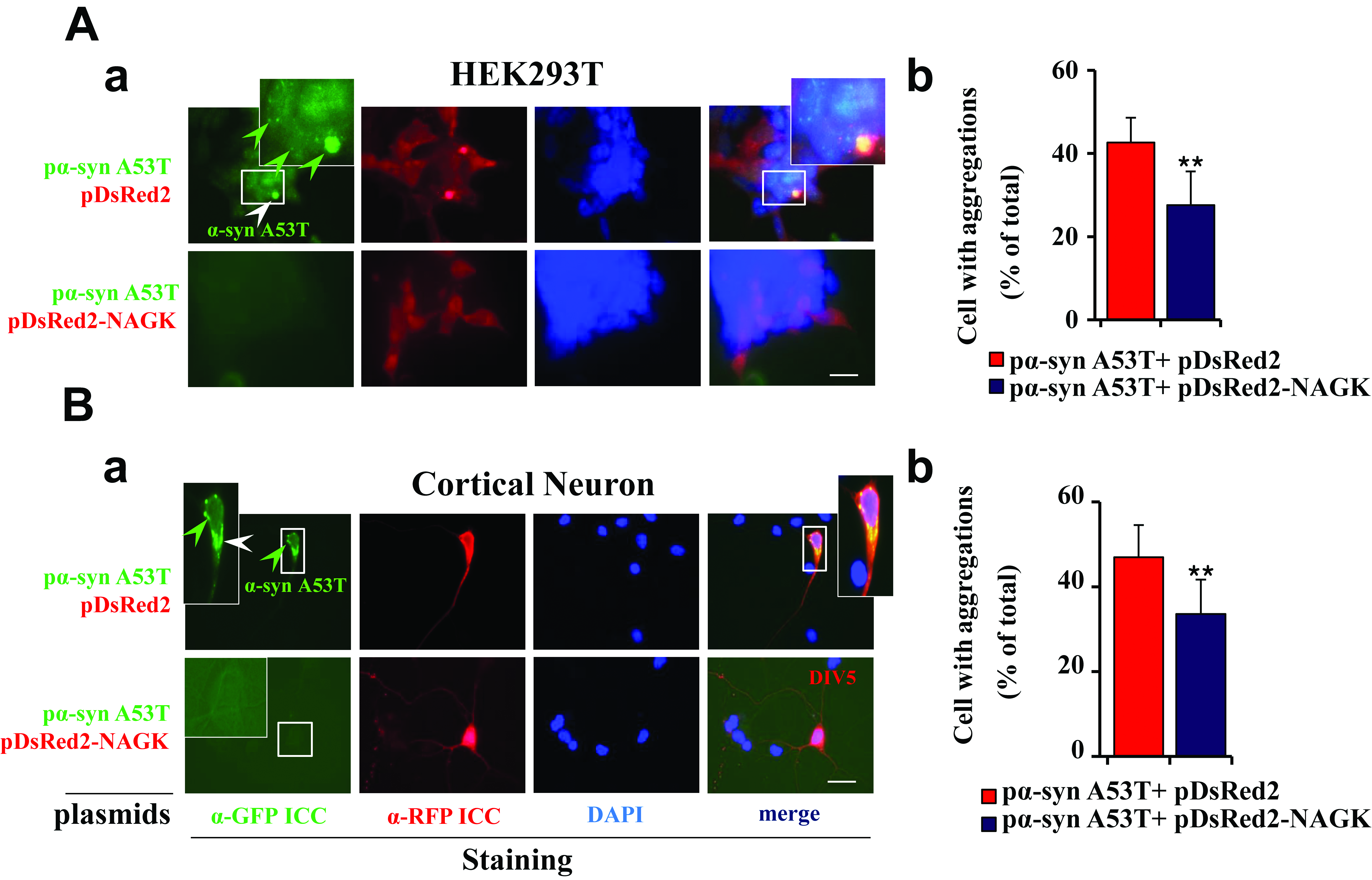

Supplement: Supplementary file 2 — Figure S1. [file 41419_2020_2862_MOESM2_ESM.tif]

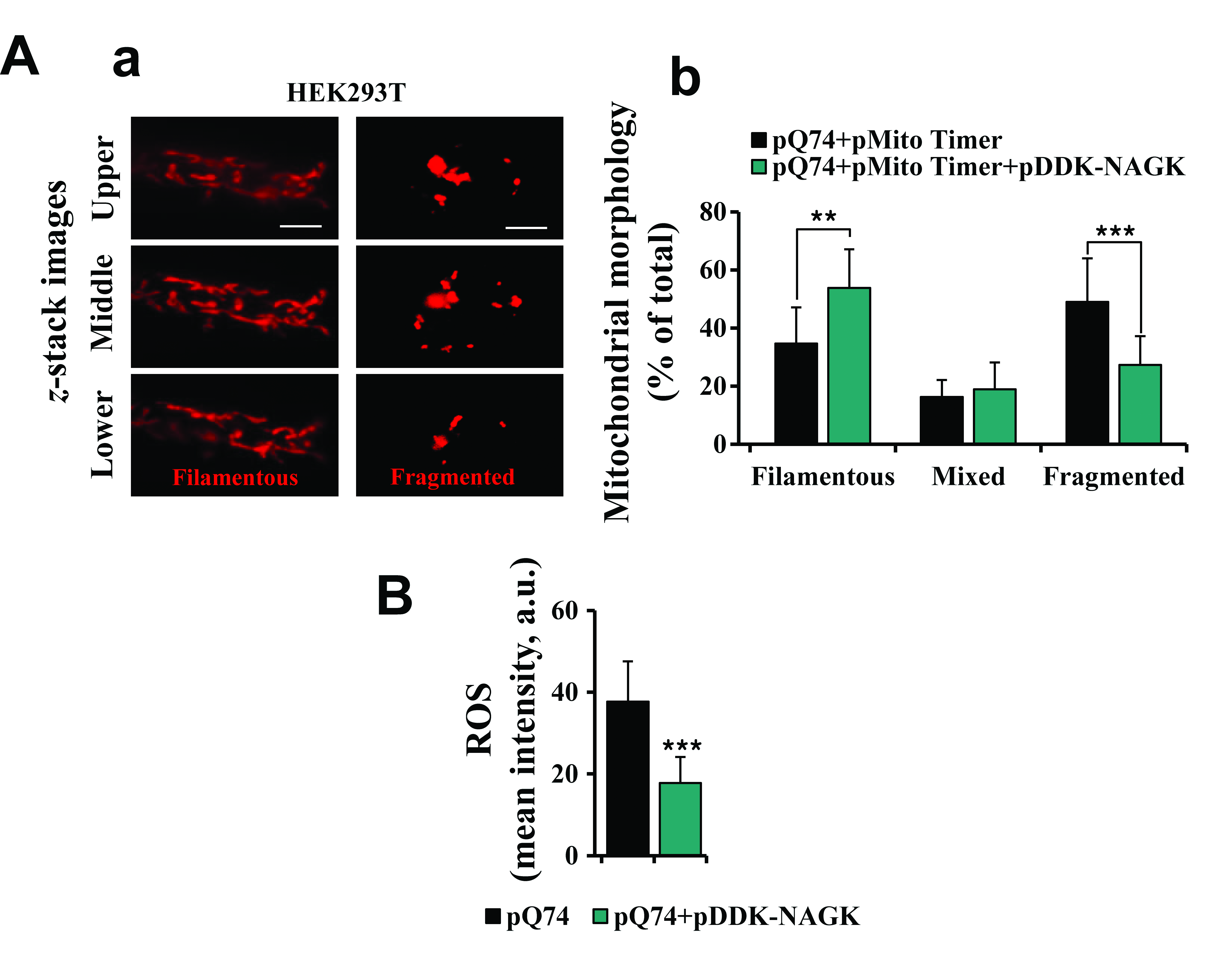

Supplement: Supplementary file 3 — Figure S2. [file 41419_2020_2862_MOESM3_ESM.tif]

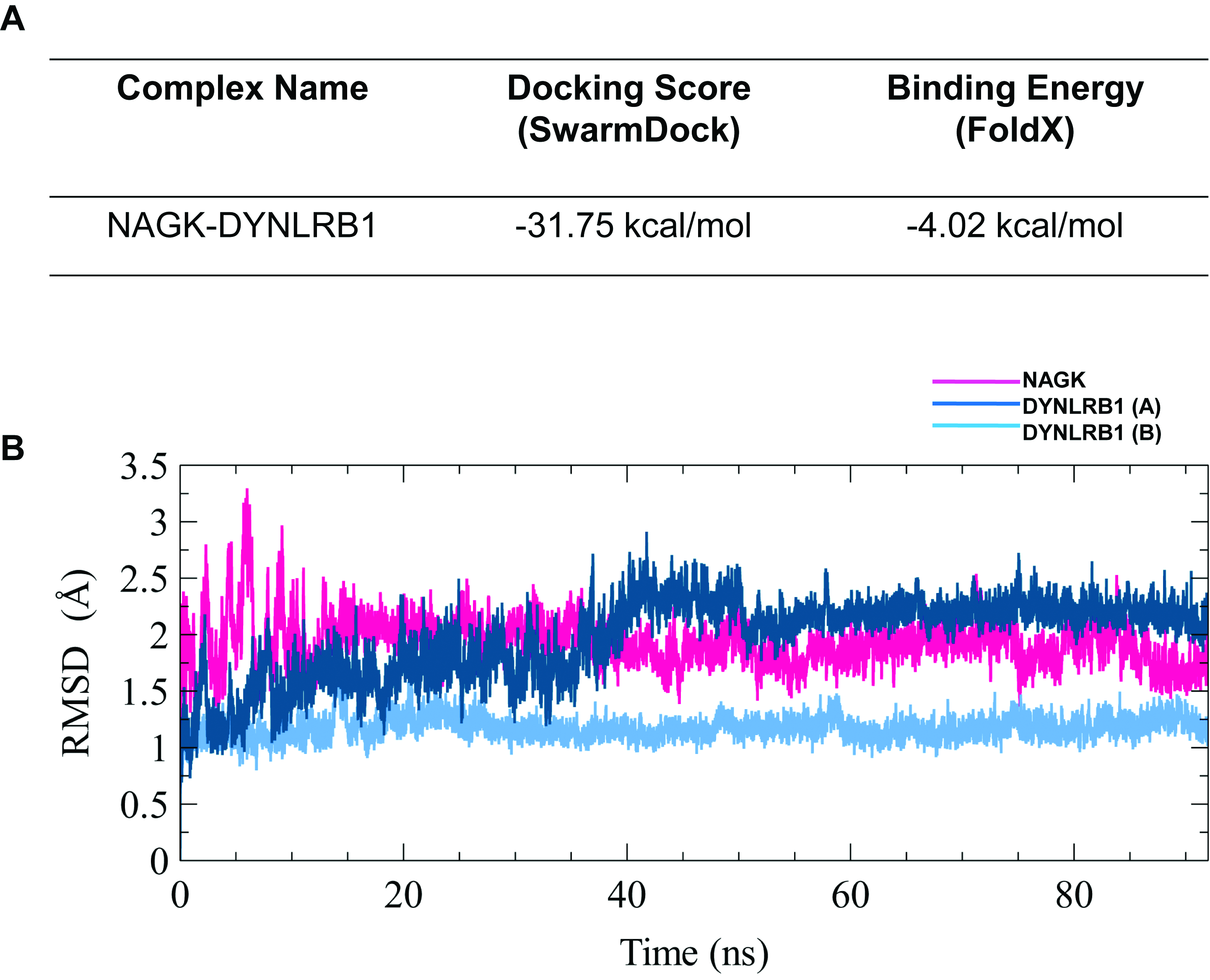

Supplement: Supplementary file 4 — Figure S3. [file 41419_2020_2862_MOESM4_ESM.tif]
